# Intracardiac Projectile

**Published:** 1918-09

**Authors:** 


**Intracardiac Projectile.** Hartmann, Le Prog. Med., Feb. 9, reports the successful extraction of a ball from just above the apex, the patient having had a pulse of 140.

---

| Period of observation. | Extremes | Ordinary range | General average |
|---|---|---|---|
| Beginning of evacuation | 1-5 min. | 5-10 | 9-10 |
| Duration of evacuation stage | 1½-3½ hrs. | 2-2½ | 2½ hrs. |
| End of evacuation | 1½-3½ | 2-2½ | 2½ |
| First appearance of bolus in colon | 1-3 | 1½-2 | 1 hr. 52 min. |
| Duration of transit of small intestine, by first bolus, | | | |
| Duration of transit of small intestine by last bolus | ½-2 hrs. | ½-1 | 1 hr. |
| Duration of evacuation period in small intestine | ¾-2 hrs. | 1½ | 1 hr. 25 min. |
| Duration of transit through small intestine of whole meal | 1¾-4½ hrs. | 3-3½ | 3¼ hrs. |
| Time after which the whole meal is collected in colon | 2-4 hrs | 3-3½ | 3-½ hrs. |

1. The beginning of gastric evacuation is scarcely influenced by small doses of papaverine—20 m.g. per kilo., is retarded by larger doses, by 30 m. g. more than by 50.

2. With all three doses, the evacuation proceeds more slowly than normal, the delay increasing with the dose.

3. The appearance of the first bolus in the colon is, perhaps, a little more rapid with the 20 m.g. dose, but delayed by the larger ones.

4. The same remark obviously applies to transit of the small intestines.

5. In all dogs to which papaverine was given, the calibre of the small intestine was reduced and there was intense peristalsis.

